# Generative AI Adoption in Psychotherapy Practice Among Mental Health Professionals: Multicountry Cross-Sectional Survey

**DOI:** 10.2196/105052

**Published:** 2026-09-30

**Authors:** Charlotte Blease, Josefin Hagström, Jens Gaab, Alexis Carey, Francesco Cipriani, Colin Gorman, Antje Frey Nascimento, Amanda Fitzgerald, Lena Holtz, Julian Schwarz, Gillian Strudwick, Maria Tibbs, John Torous, Jeffrey H D Cornelius-White

**Affiliations:** 1 Participatory eHealth and Health Data Research Group Department of Women's and Children's Health Uppsala University Uppsala, Uppsala Sweden; 2 Division of Digital Psychiatry Beth Israel Deaconess Medical Center Harvard Medical School Boston, MA United States; 3 Centre for Primary Care and Health Services Research Faculty of Biology, Medicine and Health University of Manchester Manchester, England United Kingdom; 4 Division of Clinical Psychology and Psychotherapy Faculty of Psychology University of Basel Basel Switzerland; 5 RehabCare Parkwest Dublin Dublin Ireland; 6 Department of Philosophy University College London London, England United Kingdom; 7 School of Psychology University of Ulster Coleraine, Northern Ireland United Kingdom; 8 School of Psychology University College Dublin Dublin, Leinster Ireland; 9 Department of Psychiatry and Psychotherapy Immanuel Hospital Rüdersdorf Brandenburg Medical School Rüdersdorf Germany; 10 Centre for Addiction and Mental Health Toronto, ON Canada; 11 Institute of Health Policy, Management and Evaluation University of Toronto Toronto, ON Canada; 12 Psychology in Education Research Centre Department of Education University of York York, England United Kingdom; 13 School of Mental Health and Behavioral Sciences Missouri State University Springfield, MO United States

**Keywords:** artificial intelligence, AI, generative artificial intelligence, generative AI, psychotherapy, mental health, clinical practice, large language models, digital health, survey

## Abstract

**Background:**

Generative AI (GenAI) tools, such as OpenAI’s ChatGPT, Google’s Gemini, and Microsoft Copilot, are being adopted across health care settings. Despite the popularity of GenAI, empirical data on the extent of adoption by mental health clinicians in routine psychotherapy practice are scarce.

**Objective:**

This study aimed to characterize purposeful GenAI use among mental health professionals providing psychotherapy, including tools used, purposes, perceived workload effects, and the organizational context surrounding use.

**Methods:**

We conducted a cross-sectional online survey between January 13, 2026, and March 30, 2026. A convenience sample of trained mental health professionals who had provided psychotherapy within the previous 12 months was recruited through professional associations and academic and clinical mailing lists, with snowball dissemination by email. The survey assessed lifetime purposeful GenAI use in psychotherapy practice; tools and purposes of use; perceived work burden reduction; and, for the previous 12 months, employer or professional organization encouragement, prohibition, and participation in AI training. Descriptive statistics were calculated. Univariable logistic regression models were used to examine associations between GenAI use and candidate factors. Variables meeting an entry criterion of *P*<.10 were entered into a multivariable logistic regression; statistical significance was set at *P*<.05.

**Results:**

A total of 766 mental health professionals who provide psychotherapy in 30 countries completed the survey. Of these, 54.6% (n=418, 95% CI 51.0%-58.1%) reported having purposely used at least 1 GenAI tool in clinical psychotherapy practice. ChatGPT was the most frequently used tool (354/418, 84.7%, 95% CI 81.2%-88.2%). The most commonly reported clinical purpose was assisting with treatment planning (175/418, 41.9%, 95% CI 37.1%-46.7%), followed by managing administrative tasks (n=173, 41.4%, 95% CI 36.6%-46.1%) and generating psychoeducational materials for clients (n=166, 39.7%, 95% CI 35.0%-44.4%). Among AI users, 82.8% (346/418, 95% CI 79.1%-86.4%) reported that these tools reduced their overall work burden. Only 18.1% (139/766, 95% CI 15.4%-20.9%) of respondents reported institutional encouragement to use GenAI tools, while 81.1% (n=621, 95% CI 78.3%-83.9%) reported not having received any professional training on AI use. In multivariable logistic regression (α=.05), younger age was associated with GenAI use, while practice setting showed mixed evidence of association. The overall adjusted effect of practice setting did not reach statistical significance (*P*=.07), although urban practitioners had lower odds of GenAI use than rural practitioners (adjusted odds ratio 0.614, 95% CI 0.388-0.971; *P*=.04).

**Conclusions:**

This study extends previous predominantly national and profession-specific research by providing a multicountry, multiprofessional snapshot of reported GenAI use in psychotherapy practice. Use extended beyond administrative tasks into clinically consequential activities, including treatment planning and psychoeducation, while participation in professional AI training remained relatively uncommon. Findings highlight the need for professional bodies, health systems, and training programs to develop practical guidance and competencies for safe, privacy-conscious, and clinically appropriate GenAI use.

## Introduction

### Background

The emergence of generative AI (GenAI), defined here as AI systems capable of producing text, summaries, or other content in response to natural language prompts, has precipitated rapid and largely unregulated adoption across health care professions [1-4]. Platforms such as OpenAI’s ChatGPT, Google’s Gemini, Claude AI, and Microsoft Copilot are now accessible to clinicians and patients alike, with minimal technical barriers to entry [5]. In parallel, ambient scribe tools that incorporate GenAI and passively capture and process clinical conversations in real time have begun to enter clinical workflows, raising distinct questions about consent, accuracy, and relational dynamics [6].

Mental health practice presents a particular case study in GenAI adoption. Large language models (LLMs) are experienced by some users as a relationship rather than a tool: emerging empirical work documents the formation of parasocial bonds [7-9]. Indeed, unlike many clinical approaches, psychotherapy is fundamentally relational: positive regard, therapeutic alliance, empathic attunement, and client-centered responsiveness are not incidental features but central mechanisms of effective treatment [10,11]. In addition, practitioners working in psychotherapy contexts also face significant documentation and administrative burdens that GenAI tools may be able to meaningfully alleviate [8].

A 2024 systematic review similarly found that research on LLMs in mental health was dominated by studies of model performance, screening, and conversational applications, with limited evaluative evidence supporting their use in clinical settings and important gaps in clinical integration, privacy, interpretability, and ethical governance [12]. Yet comparatively little is known about how mental health professionals themselves are actually incorporating GenAI into psychotherapy practice.

Prior surveys of AI attitudes and behaviors among health professionals have predominantly sampled physicians in high-income countries [13-15]. In the era of GenAI, surveys of mental health professionals specifically are nascent. However, in North America, this is beginning to change. In 2025, 56% of American Psychological Association psychologists reported using these tools in practice [16]. A study comparing German and US practitioners similarly found substantial familiarity gaps with AI applications across diagnosis, treatment, feedback, and practice management, with learning intention, ethical knowledge, and technology affinity emerging as cross-domain predictors of adoption intentions [17]. Another qualitative interview study of 18 US psychotherapists found that trust in GenAI was conditional and task-dependent, sustained for low-stakes administrative work such as documentation and brainstorming, but withdrawn when AI threatened clinician control or clinical judgment [18]. A related multinational study, including German clinicians, similarly described a mix of enthusiasm and skepticism, framing clinicians’ evaluation of GenAI in terms of epistemic trust and a “hybrid therapeutic alliance,” with concerns centered on response quality, the therapeutic relationship, and data ethics [19].

Despite these emerging studies, comparative and multicountry evidence remains limited, particularly regarding the breadth of GenAI use across mental health professions, including tools employed, purposes served, perceived workload impact, and the organizational context surrounding use and training. This study addresses that gap. Drawing on a global convenience sample of practicing mental health professionals, we characterize the landscape of GenAI adoption in clinical psychotherapy practice in the first quarter of 2026. Our findings are intended to inform policymakers, professional bodies, and training programs seeking to develop evidence-based responses to GenAI integration in mental health services.

### Research Objectives

The research objectives are as follows:

- To determine the prevalence of purposeful GenAI use among mental health professionals globally
- To identify the specific GenAI tools in use and the clinical and administrative purposes they serve
- To assess whether clinicians perceive GenAI tools as reducing their work burden
- To characterize the institutional context of GenAI use, including employer encouragement, prohibition, and participation in formal training
- To examine demographic and professional factors associated with GenAI adoption

## Methods

### Study Design

This was a cross-sectional mixed methods survey conducted between January and March 2026. The survey was administered online via LimeSurvey (LimeSurvey GmbH). This paper reports the quantitative survey findings from sections A (AI use behaviors) and D (demographics) of a larger questionnaire (for the full survey instrument, refer to Multimedia Appendix 1). This study was conducted and reported in accordance with the CHERRIES (Checklist for Reporting Results of Internet E-Surveys) checklist for online health surveys [20] (Multimedia Appendix 2) and the APA Journal Article Reporting Standards for Quantitative Research (JARS-Quant; Multimedia Appendix 3).

### Ethical Considerations

Ethics approval was obtained from the institutional review board of the Faculty of Psychology, University of Basel (017-25-1) and the Missouri State University Institutional Review Board (IRB-FY2025-325). All participants provided informed electronic consent prior to survey commencement. Participation was voluntary, anonymous, and unpaid. Personally identifying information—including names, contact details, and IP addresses—was not collected. LimeSurvey is a secure online platform that uses encryption and anonymization to prevent survey responses from being linked to individual participants. The platform operates in compliance with the European Union General Data Protection Regulation (GDPR). No images depicting individuals were collected or are included in the manuscript or multimedia appendices.

### Participants and Eligibility

Eligible participants were trained mental health professionals—including psychotherapists, psychologists (clinical and counseling), counselors, psychiatrists incorporating psychotherapy, social workers providing psychotherapy, nurses providing psychotherapy, and other self-identified practitioners of psychotherapy—who were currently practicing or had practiced within the prior 12 months. Participants were required to be aged ≥18 years and to provide informed consent electronically. There were no country-of-practice restrictions.

### Recruitment

Participants were recruited via a global convenience sampling strategy between January 13, 2026, and March 30, 2026 (approximately 11 weeks). As this was an online multicountry survey, there was no single clinical recruitment site. Participants were recruited from professional networks and organizations spanning a range of psychotherapy practice settings. Recruitment used convenience and snowball sampling through professional associations, academic and clinical mailing lists, and direct professional contacts. The survey link was disseminated directly and through snowball-sampling requests to professional contacts via email. Recruitment channels included professional associations and networks (psychology, psychiatry, counseling, and social work bodies), as well as academic and clinical mailing lists predominantly in Austria, Canada, Ireland, Germany, Switzerland, the United Kingdom, and the United States. We deliberately avoided using all social media platforms to administer the survey to avoid the risk of bot contamination. To preserve respondent anonymity, IP addresses, cookies, or registration identifiers were not collected, and no time stamp–based duplicate response screening was undertaken. Conditional branching was used for selected items. The minimum target sample size was calculated a priori using a precision-based sample size calculation for a single proportion (anticipated AI use prevalence=30%; margin of error=5%; 95% CI; N≈323), with a target of ≥500 completed responses to enable subgroup analyses. Because the study used convenience and snowball sampling, this calculation was used to inform recruitment targets and should not be interpreted as providing population-level precision or addressing selection bias.

### Survey Instrument

The survey was developed iteratively by the lead members of the research team (CB, JHDC-W, and JG) with expertise in digital mental health, clinical psychology and psychotherapy, and survey methodology. The English-language questionnaire was pretested with 3 clinical psychologists to assess clarity, comprehensibility, and clinical relevance. The questionnaire was subsequently translated into German, and the German-language version was reviewed by 2 native German-speaking clinical psychologists for linguistic accuracy, comprehensibility, and appropriate clinical terminology before fielding. The German-language instrument is provided in Multimedia Appendix 1.

The survey was timed to take approximately 5 to 7 minutes to complete and included 4 sections—section A encompassed GenAI adoption (as described below), section B included items focusing on participants’ opinions about GenAI in psychotherapy, section C included an open comment question, and section D focused on demographic characteristics. The questionnaire was presented grouped by section and consisted of 5 survey sections or pages. All closed-ended items were mandatory, while 1 open-ended free-text question was optional. For this paper, the following sections of the survey are reported (Table 1; Multimedia Appendix 1). Free-text responses entered under “Other” for tools and purposes of use were reviewed and grouped descriptively into recurring categories; no formal qualitative analysis of the separate open-ended section C responses is reported in this paper. We note that in the study we inquired about participants’ “purposeful” use of GenAI, which referred to conscious, deliberate use of GenAI tools to assist with psychotherapy practice; the item was not designed to capture passive or unrecognized use of embedded GenAI functionality.

**Table 1:** Survey sections reported in this manuscript from a cross-sectional, multicountry survey of mental health professionals providing psychotherapy (January 2026-March 2026; N=766).

Sections and questions			Response options
**Section A: AI use behaviors**			
	Purposeful AI use	Yes and no	
	Tools used	ChatGPT, Microsoft Copilot or Bing AI, Bard or Gemini, Claude, Perplexity, Consensus, HEIDI, and other	
	Purposes of use	Session notes, psychoeducational materials, treatment planning, reviewing or summarizing notes, administrative tasks, communication with colleagues, reflective practice or supervision, and other (multiselect)	
	Perceived burden reduction	Yes and no	
	Employer or professional organization encouragement	Yes, no, and do not know	
	Employer or professional organization prohibition	Yes, no, and do not know	
	Participation in professional training on AI	Yes, no, and do not know	
**Section D: demographics**			
	Professional role	Psychotherapist (public), psychotherapist (private), psychotherapist in a university or academic setting, psychotherapist in a nonprofit or nongovernmental organization, clinical psychologist, counseling psychologist, counselor, psychiatrist, social worker providing psychotherapy, mental health nurse providing psychotherapy, self-employed in mental health services, other psychologist, and other (multicategory)	
	Therapy formats currently used	In-person or face-to-face sessions, phone- or video-based sessions (teletherapy), asynchronous communication (eg, email, messaging, and chat), and digital tools or platforms to support therapy (eg, apps, online exercises, automated check-ins, and AI tools; multiselect)	
	Primary delivery modality in past 3 months	In-person, teletherapy, mixed, and other	
	Gender	Man, woman, prefer not to say, and other	
	Age	Continuous	
	Country of practice	Free text	
	Practice area type	Urban, suburban, and rural	

### Statistical Analysis

Data were exported from LimeSurvey. Only completed questionnaires were analyzed. Descriptive statistics are reported as frequencies and percentages for categorical variables and means with SDs for continuous variables (age).

Univariable binary logistic regression models were used to examine associations between AI use (yes or no) and candidate factors (age, gender, professional role, primary delivery modality, practice setting, and country income group [World Bank classification]) and to obtain unadjusted odds ratios (ORs) with 95% CIs. Variables meeting an entry criterion of *P*<.10 were entered into a multivariable binary logistic regression model. This prespecified approach was used to identify candidate factors associated with GenAI use while limiting model complexity and the number of parameters included in the final model. Univariable logistic regression models were fitted separately for each candidate variable; sample sizes varied slightly across these models because respondents in very small categories (eg, gender: other or prefer not to say; modality: other) and conceptually heterogeneous groups (eg, profession: other) were excluded from the regression analyses for that variable. Results are reported as adjusted ORs (aORs) with 95% CIs and 2-sided *P* values; the significance threshold was set at *P*<.05. Analyses were conducted in SPSS (version 30.0; IBM Corp).

## Results

### Sample Characteristics

Participant flow is shown in Figure 1. A total of 766 participants completed the survey (completion rate: 72.6% of those who opened the survey link [N=1055]). Respondents were located across 30 countries, predominantly from Germany (402/766, 52.5%) and the United States (n=87, 11.4%). Other reported countries were Argentina, Australia, Austria, Belgium, Brazil, Bulgaria, Canada, Chile, Colombia, Denmark, Finland, France, Greece, Hungary, Ireland, Israel, Italy, Japan, the Netherlands, New Zealand, Portugal, Romania, Slovenia, Spain, Sweden, Switzerland, the United Kingdom, and Ukraine. A full list of countries and detailed demographic and professional characteristics of the sample are presented in Table 2.

### Prevalence and Tools Used

Overall, 418 of 766 (54.6%, 95% CI 51.0%-58.1%) respondents reported having purposely used at least 1 GenAI tool in their psychotherapy practice. Among these 418 users, ChatGPT (OpenAI) was the most frequently used tool (n=354, 84.7%, 95% CI 81.2%-88.2%), followed by Google Bard or Gemini (Alphabet Inc; n=74, 17.7%, 95% CI 14.0%-21.4%) and Microsoft Copilot or Bing AI (Microsoft Corp; n=53, 12.7%, 95% CI 9.5%-15.9%). “Other” free-text responses primarily included VIA Health (Cardon Outreach LLC; 4.3%), Mistral Le Chat or Vibe (Mistral AI; 1.7%), and Grok (SpaceXAI; 0.7%). Complete data on tool use frequencies are presented in Table 3, and all free-text responses are provided in Table S1 in Multimedia Appendix 4.

**Figure 1:**
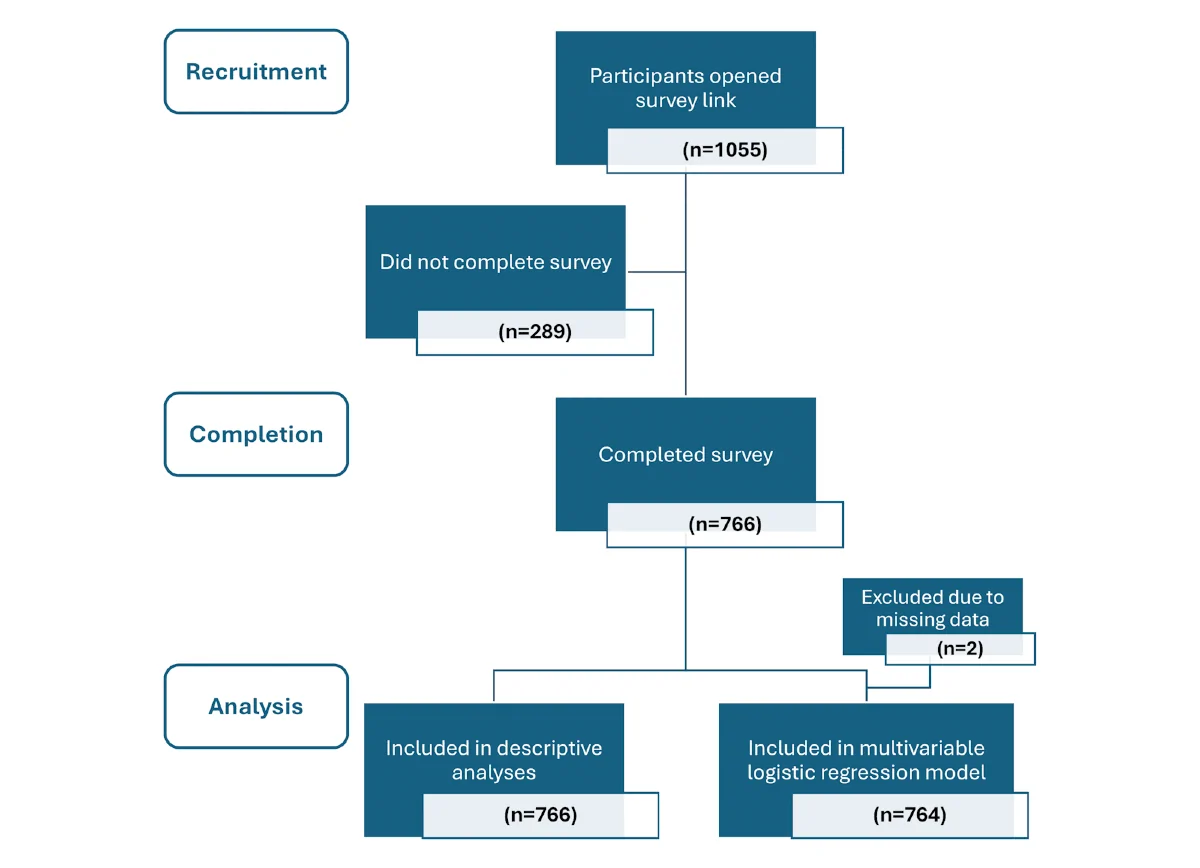
Study flowchart showing participant recruitment, survey completion, and analysis across study phases.

**Table 2:** Demographic and professional characteristics of the sample (N=766).

Characteristics			Participants
**Gender, n (%)**			
	Women	510 (66.6)	
	Men	235 (30.7)	
	Other or prefer not to say	16 (2.1)	
	Missing	5 (0.7)	
Age (years), mean (SD; range)			47.81 (14.03; 22-89)
**Country, n (%)**			
	Germany	402 (52.5)	
	United States	87 (11.4)	
	Ireland	53 (6.9)	
	Switzerland	49 (6.4)	
	United Kingdom	33 (4.3)	
	Canada	25 (3.3)	
	Greece	23 (3)	
	Austria	14 (1.8)	
	Denmark	12 (1.6)	
	Romania	11 (1.4)	
	Belgium	9 (1.2)	
	Argentina	5 (0.7)	
	Australia	5 (0.7)	
	Portugal	5 (0.7)	
	France	3 (0.4)	
	Israel	3 (0.4)	
	Japan	3 (0.4)	
	Spain	3 (0.4)	
	Ukraine	3 (0.4)	
	Brazil	2 (0.3)	
	Chile	2 (0.3)	
	Sweden	2 (0.3)	
	Italy	1 (0.1)	
	Bulgaria	1 (0.1)	
	Colombia	1 (0.1)	
	Finland	1 (0.1)	
	Hungary	1 (0.1)	
	Netherlands	1 (0.1)	
	New Zealand	1 (0.1)	
	Slovenia	1 (0.1)	
	Missing	4 (0.5)	
**Professional role, n (%)**			
	Psychotherapist (public)	194 (25.3)	
	Psychotherapist (private)	187 (24.4)	
	Clinical psychologist	81 (10.6)	
	Psychotherapist in a university or academic setting	53 (6.9)	
	Counselor	53 (6.9)	
	Counseling psychologist	27 (3.5)	
	Psychiatrist	24 (3.1)	
	Psychotherapist in a nonprofit or nongovernmental organization	14 (1.8)	
	Other^a^	133 (17.4)	
**Primary delivery modality (past 3 months), n (%)**			
	Mostly in-person (≥75%)	619 (80.8)	
	Mostly teletherapy (≥75%)	72 (9.4)	
	Mixed (≈40%-60% split)	54 (7)	
	Other	21 (2.7)	
**Practice setting, n (%)**			
	Urban	535 (69.8)	
	Suburban	134 (17.5)	
	Rural	97 (12.7)	
**World Bank country income group, n (%)**			
	High income	751 (98)	
	Upper middle income	11 (1.4)	
	Lower middle or low income	0 (0)	
	Missing	4 (0.5)	

^a^“Other” responses for professional role included mainly (1) professionals working in multiple roles or settings (eg, private practice and academic, public health care and nongovernmental organization), (2) professions not listed among the response options (eg, occupational therapists, nurses, and physicians), and (3) trainees (eg, individuals in psychotherapy, psychology, or counseling programs). Specific roles included medical physician, physician psychotherapist, self-employed in mental health services, social worker providing psychotherapy, mental health nurse providing psychotherapy, and student or trainee.

**Table 3:** Generative AI tools used by mental health professionals who indicated use in clinical practice (N=418).

Tools	AI users, n (%)^a^
ChatGPT (OpenAI)	354 (84.7)
Google Bard or Gemini	74 (17.7)
Microsoft Copilot or Bing AI	53 (12.7)
Perplexity	43 (10.3)
Claude (Anthropic PBC)	27 (6.5)
VIA Health	18 (4.3)
HEIDI Health	8 (1.9)
Consensus	8 (1.9)
Other (specify)	70 (16.7)

^a^Percentages sum to >100% because respondents could select multiple generative AI tools.

### Purposes of GenAI Use

Among 418 GenAI users, assisting with treatment planning was the most commonly endorsed purpose (n=175, 41.9%, 95% CI 37.1%-46.7%), followed by managing administrative tasks (n=173, 41.4%, 95% CI 36.6%-46.1%). Reviewing and summarizing prior client notes (n=72, 17.2%, 95% CI 13.6%-20.9%) and enhancing communication with colleagues (n=69, 16.5%, 95% CI 12.9%-20.1%) were reported less frequently. Those who selected “other” (n=94, 22.5%, 95% CI 18.5%-26.5%) described a variety of additional uses, the main ones being text production and language, information search, professional reporting, clinical reasoning, and teaching or training. Full data are presented in Table 4, and subthemes and frequencies for the free-text response themes are found in Table S2 in Multimedia Appendix 4.

### Perceived Workload Impact

Among those who had used GenAI tools, 82.8% (346/418, 95% CI 79.1%-86.4%) reported that these tools had reduced their work burden overall, and 17.2% (n=72, 95% CI 13.6%-20.9%) reported that GenAI tools had not reduced their work burden.

**Table 4:** Purposes of generative AI tool use reported by mental health professionals who indicated use in clinical practice (N=418).

Purposes	AI users, n (%)
Assisting with treatment planning	175 (41.9)
Managing administrative tasks	173 (41.4)
Generating psychoeducational materials for clients	166 (39.7)
Supporting reflective practice or supervision	136 (32.5)
Session note documentation	128 (30.6)
Reviewing and summarizing prior client notes	72 (17.2)
Enhancing communication with colleagues	69 (16.5)
Other	94 (22.5)

### Institutional Context: Encouragement, Prohibition, and Training

Across all respondents, 18.1% (139/766, 95% CI 15.4%-20.9%) reported that their employer or professional organization had encouraged them to use GenAI tools in the past 12 months, while 12.7% (n=97, 95% CI 10.3%-15.0%) indicated they were unaware of any such encouragement. In contrast, 8.2% (n=63, 95% CI 6.3%-10.2%) reported that their organization had prohibited AI use. Formal professional training or workshops on GenAI were reported by only 18.3% (n=140, 95% CI 15.5%-21.0%) of respondents, while 81.1% (n=621, 95% CI 76.3%-83.9%) reported no such training, and 0.7% (n=5, 95% CI 0.1%-1.2%) reported not knowing whether they had received such training. As shown in Figure 2, respondents were substantially more likely to report not receiving encouragement or training related to GenAI than to report receiving such support.

**Figure 2:**
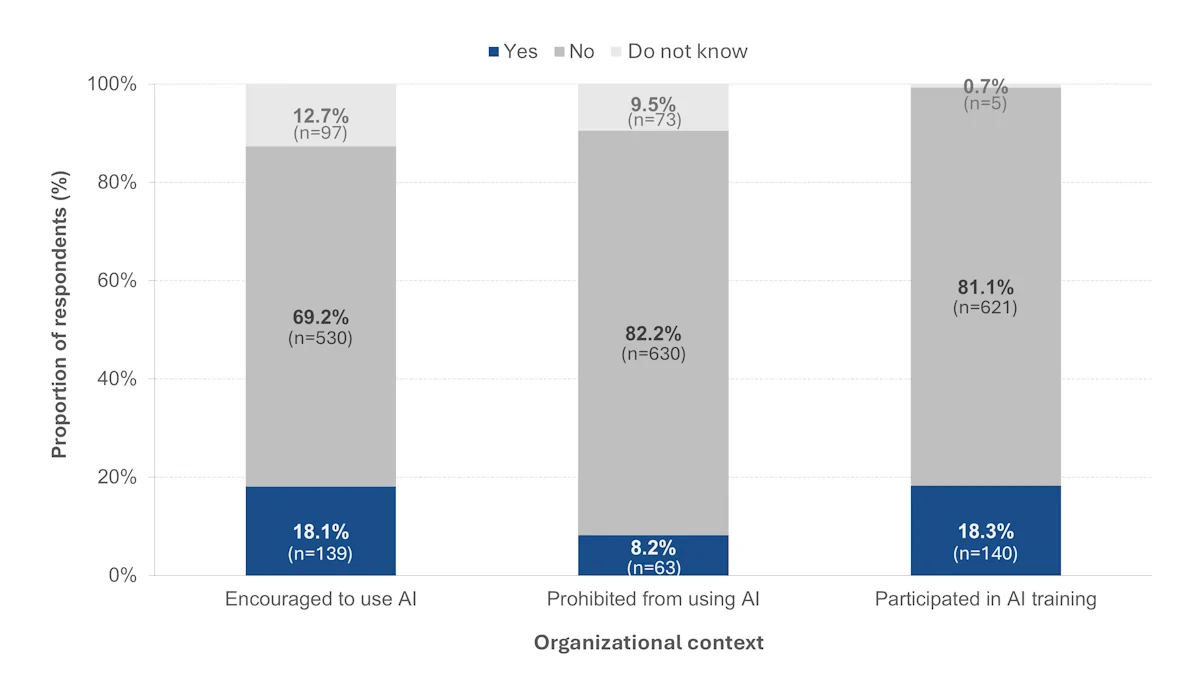
Distribution of respondents according to employer or professional organization encouragement or prohibition of AI use and participation in professional AI-related training during the previous 12 months among mental health professionals providing psychotherapy (N=766).

### Factors Associated With GenAI Use

Overall, younger age was associated with reported GenAI use, and practice setting showed a similar pattern in unadjusted analysis; both associations were modest in strength. In univariable analysis, GenAI use was significantly associated with age (*P*<.001) and practice setting (*P*=.049). In the multivariable logistic regression model, factors independently associated with GenAI use were younger age (aOR 0.976, 95% CI 0.966-0.986; *P*<.001) and practice setting. Compared with those who reported they were rural practitioners, those in urban settings (aOR 0.614, 95% CI 0.388-0.971; *P*=.04) and suburban settings (aOR 0.537, 95% CI 0.311-0.930; *P*=.03) had significantly lower odds of GenAI use. The overall adjusted effect of practice setting did not reach statistical significance (*P*=.07), although urban and suburban practitioners had lower odds of GenAI use than rural practitioners in individual contrasts. In our sample, professional role, primary modality, gender, and country income group were not entered into the multivariable model, as they did not meet the screening criterion of *P*<.10 in univariable analyses. The final model explained a modest proportion of variance in GenAI use (Nagelkerke *R*^2^=.047). Full regression results are presented in Table 5.

**Table 5:** Univariable and multivariable logistic regression: factors associated with purposeful generative AI use among mental health professionals^a^.

Variables			OR^b^ (95% CI)		*P* value			Adjusted OR (95% CI)			*P* value	
Age (years; n=764)			0.976 (0.965-0.986)		<.001			0.976 (0.966-0.986)			<.001	
Gender (reference: man; n=745)			1.221 (0.895-1.665)		.21			—^c^			—	
**Professional role (reference: private psychotherapist; n=633)**						.31			—			—
	Psychotherapist employed by a public health care system	1.322 (0.880-1.986)		.18			—			—		
	Psychotherapist in a university or academic setting	0.604 (0.326-1.120)		.11			—			—		
	Psychotherapist in a nonprofit or nongovernmental organization	0.851 (0.287-2.523)		.77			—			—		
	Counselor	0.820 (0.445-1.510)		.52			—			—		
	Clinical psychologist	1.119 (0.662-1.892)		.68			—			—		
	Counseling psychologist	1.448 (0.630-3.327)		.38			—			—		
	Psychiatrist incorporating psychotherapy in practice	1.192 (0.504-2.820)		.69			—			—		
**Setting (reference: rural; n=766)**						.049						.07
	Urban	0.592 (0.376-0.932)		.02			0.614 (0.388-0.971)			.04		
	Suburban	0.531 (0.310-0.911)		.02			0.537 (0.311-0.930)			.03		
**Modality (reference: mostly in-person [≥75% of sessions face-to-face]; n=745)**						.64			—			—
	Mostly teletherapy: live phone or video (≥75% remote)	1.127 (0.687-1.847)		.64			—			—		
	About an even mix (≈40%-60% split between in-person and teletherapy)	0.805 (0.461-1.404)		.44			—			—		
Country income group (n=762)			0.998 (0.302-3.299)		>.99			—			—	

^a^Sample sizes differed across analyses because some categories were excluded due to small cell sizes; the professional role “other” category was excluded because it was heterogeneous and not comparable with other categories. The multivariable model was estimated on 764 respondents, with 2 respondents excluded owing to missing age data. Nagelkerke *R*^2^=0.047.

^b^OR: odds ratio.

^c^Variable not entered into multivariable model because it did not meet the screening criterion of *P*<.10.

## Discussion

### Principal Findings

This global survey of 766 mental health professionals characterizes the breadth and context of GenAI adoption in clinical psychotherapy practice across multiple countries. Our central finding, that approximately 54.6% of respondents reported having used GenAI tools in clinical practice, suggests that adoption may be substantial in this professionally and geographically diverse convenience sample. However, these findings should be interpreted in light of the sample composition, particularly the predominance of respondents from Germany and other high-income settings. It is also unclear if use reflected one-off experimentation or habitual use of GenAI. We note that the reported rate, nonetheless, is consistent with, and in some analyses exceeds, GenAI use estimates from concurrent surveys of physicians and clinical psychotherapists [2,4,16], suggesting that mental health professionals are not lagging behind other health disciplines in using these tools.

Our findings show that, among mental health professionals, younger clinicians reported greater use of GenAI tools. The higher reported GenAI use among rural practitioners was unexpected. One possibility is that clinicians in rural settings may turn to GenAI where access to colleagues, specialist consultation, or other professional resources is more limited; however, the rural subgroup was relatively small, and the CIs approached 1.0, so this exploratory finding should be interpreted cautiously and requires replication. The present study did not measure mechanisms that could explain this association.

The low explanatory power of the model indicates that the demographic and professional characteristics measured in this study explain only a small proportion of the variation in reported GenAI use. As such, the significant associations identified should be interpreted as factors associated with use within a model with limited overall explanatory power rather than as comprehensive explanations of adoption behavior.

Across the tools adopted (predominantly ChatGPT, a general-purpose LLM) and the purposes endorsed (predominantly treatment planning, managing administrative tasks, psychoeducational content, and reflective practice), what emerges is a picture of AI integration. Clinicians may be turning to AI to reduce workplace burdens, but also for core clinical support. The use of GenAI for treatment planning is particularly noteworthy because it may extend AI involvement beyond administrative support into clinically consequential decision-making. However, the survey did not capture how GenAI was incorporated into treatment planning, whether outputs were independently verified, or what information was entered into these systems; these questions warrant direct investigation in future studies. This does, however, raise important questions about how and whether these tools are effectively adopted, and how they might influence clinical accuracy and treatment outcomes [21-23].

Relatedly, adoption also raises fundamental concerns about data privacy, particularly in countries with strict data protection frameworks [24,25]. The predominance of general-purpose tools such as ChatGPT, including for treatment planning, raises important data protection and confidentiality concerns. Our survey did not assess whether clinicians entered identifiable client information, which deployment or subscription tier was used, or whether use occurred within institutionally approved systems; therefore, no conclusions can be drawn about the compliance of respondents’ individual practices.

The finding that most AI users perceived a reduction in work burden aligns with emerging data from medicine, which identifies ambient AI scribes as reducing burdens but not necessarily time spent on tasks such as documentation [26]. This finding should be interpreted as a perception among respondents who reported GenAI use, rather than as evidence that GenAI reduces workload across mental health professionals generally. The survey did not distinguish sustained users from those who may have experimented with GenAI and discontinued use, so selection effects may have contributed to the high proportion reporting reduced work burden.

For mental health professionals, who face distinctive emotional labor, burnout risks, and extensive administrative demands, even modest efficiency gains in documentation may translate into meaningful restoration of time for direct clinical care or personal recovery [6]. Qualitative studies from psychotherapy settings further suggest that clinicians experience reduced emotional and cognitive burden, greater presence during sessions, and perceived improvements in documentation quality when using AI-supported documentation tools [27-29].

More than half of respondents reported ever having purposely used GenAI in psychotherapy practice, while only a minority reported participating in professional AI training during the previous 12 months. Because these measures use different reference periods, they should not be interpreted as a direct comparison of adoption and training rates. Potential training gaps may reflect the speed with which GenAI tools have entered clinical practice relative to the development of institutional policies and professional education, potentially leaving clinicians to navigate questions of appropriate use, privacy, and clinical oversight without formal preparation. Additionally, not supporting clinicians in the adoption of GenAI in their practice may be preventing them from realizing the benefits these tools can bring to their well-being and from reducing burnout risks, as well as from potentially improving quality.

### Comparison With Existing Literature

Prior work on AI attitudes and behaviors in mental health has been limited in scope and geography [15]. US-based and German surveys found that ChatGPT adoption was high among surveyed psychiatrists [13]. Our survey is also consistent with findings from the American Psychiatric Association and American Psychological Association [4,16]. The previously mentioned German survey also reported cautious attitudes toward AI in clinical decision support, with concerns about liability and data security predominating [30]. Our findings also extend previous surveys by our group. In 2024, 20% of UK general practitioners reported using GenAI in clinical practice, while a contemporaneous survey of psychiatrists found substantial use of ChatGPT to assist with clinical questions [14]. In 2025, this rose to 25% [1], and by 2026, the figure had more than doubled to 52% [2]. Although differences in sampling, professional groups, and survey items preclude direct prevalence comparisons, together these studies suggest a rapid broadening of GenAI use across clinical professions and an increasing role for these tools in both administrative and clinically consequential tasks.

Our data extend these findings to a global, multiprofessional sample and move beyond attitudes to characterize actual behaviors, tools, purposes, and institutional context. The dominance of ChatGPT in our sample, despite the availability of health care–specific tools, such as Nuance DAX and HEIDI, suggests that cost, accessibility, familiarity, and institutional context may drive adoption, rather than clinical suitability. Relatedly, emerging studies indicate that patients and the public are rapidly adopting GenAI tools for mental health and emotional support, in particular those with no health insurance, minorities, and younger people [31,32]. These studies suggest that ease of access to these tools, costs, waiting lists, and fear of stigmatization by health professionals are reasons for GenAI adoption.

### Implications for Policy and Practice

At the outset, given that a large proportion of respondents were based in European countries, these concerns are especially salient under GDPR, which places strict requirements on the processing of personal health data and therefore has direct implications for the use of GenAI tools in clinical contexts. Respondents outside Europe may be subject to different data protection, professional, and AI governance frameworks; therefore, the GDPR-related considerations discussed here may not apply uniformly across the international sample. Our findings also carry several implications for professional bodies, regulators, and training programs. First, competency frameworks for ethical and safe GenAI use in psychotherapy are urgently needed. The APA, British Psychological Society, and equivalent bodies have begun to issue guidance, but formal curricula are sparse [33,34]. Professional bodies could establish minimum GenAI competencies and incorporate them into continuing professional development. Second, the documented gap between informal adoption and institutional training or oversight represents a governance failure that needs to be addressed in licensing and continuing professional development frameworks. Third, the predominance of general-purpose, consumer LLMs over health care–specific tools in this sample suggests a need for better dissemination of information about purpose-built clinical AI options, including their regulatory status, data protection characteristics, and accuracy benchmarks.

### Limitations

There are several limitations to this study. First, this was a convenience sample recruited through professional networks and was geographically uneven. More than half of respondents practiced in Germany, and the sample was predominantly drawn from European and other high-income settings: 98% of respondents practiced in high-income countries, with none from low-income countries. The findings therefore should not be interpreted as representative of the global psychotherapy workforce and may have limited generalizability to non-Western cultural and health care contexts, where patterns of GenAI access, use, regulation, and professional practice may differ. Recruitment through professional associations, academic and clinical mailing lists, and professional networks may also have preferentially attracted clinicians with greater interest in digital health or AI, potentially inflating reported GenAI use relative to the wider professional population. A related concern is that many clinicians are already burned out and facing constrained time resources, and the additional burden of survey fatigue may have further biased the sample toward those with established interest in AI. Second, the primary outcome measured lifetime purposeful ever-use and did not capture frequency, recency, duration, or intensity of use; consequently, the study cannot distinguish one-off experimentation from routine integration into practice. Third, self-report data are also subject to social desirability, recall bias, and varying understandings of what constitutes GenAI tools.

Fourth, our survey instrument was developed in English with a German translation. Owing to the resource constraints of the survey team, these linguistic limitations and language barriers limited participation from non–English-speaking and non–German-speaking professionals and—again—this likely skewed the geographic distribution of responses. Fifth, because almost all respondents practiced in high-income countries, there was limited variability in country income group, and findings related to this variable should be interpreted cautiously. Given the relatively high prevalence of the outcome, ORs may overstate the magnitude of association relative to risk ratios and should be interpreted accordingly. Alternative model-building approaches, such as forced entry of all candidate variables, may have produced different patterns of association, and these findings should be interpreted as exploratory.

Finally, because the survey was anonymous, IP-based and other identifying checks were not used to verify unique participation. Although recruitment was restricted to professional networks and email dissemination rather than social media, duplicate submissions cannot be definitively excluded; however, we identified no evidence suggesting that this occurred.

### Future Research

Future studies should examine the clinical outcomes associated with GenAI tool use in psychotherapy, particularly whether AI-assisted documentation affects note quality, session attentiveness, or therapeutic alliance ratings. Longitudinal designs are needed to track the trajectory of adoption and the impact of emerging professional training initiatives. A fruitful future research study would be to understand whether this is due to younger clinicians feeling more comfortable with the technology, or due to a lack of established efficient practices that come with more experience [35]. Qualitative exploration of the reasons practitioners endorsed or avoided AI—including ethical reasoning, institutional norms, and practical barriers—would complement the quantitative profiling provided here. Specific investigation of GenAI use in low- and middle-income country contexts, where mental health workforce shortages are most acute, should be a priority. Future studies should also examine factors not measured in the present survey that may contribute to GenAI adoption, including digital literacy, previous experience with AI tools, attitudes toward technology, perceived usefulness, workload pressures, organizational culture, and institutional policies. Finally, although out of the scope of the present study, but necessary to complement our findings, we strongly advocate for much greater research attention on client use of GenAI tools as emotional support tools, both as adjuncts to, and as replacements for, mental health clinicians.

### Conclusions

This study extends previous predominantly national and profession-specific research by providing a multicountry, multiprofessional snapshot of reported GenAI use in psychotherapy practice. Our findings indicate that use is not confined to administrative support but extends into activities that may influence therapeutic content and clinical decision-making, including treatment planning and psychoeducation. At the same time, participation in professional AI training remains relatively uncommon. These findings provide an empirical benchmark for the field and highlight the need for professional bodies, health systems, and training programs to develop practical guidance and competencies for safe, privacy-conscious, and clinically appropriate GenAI use. Future research should examine how these uses affect clinical quality, privacy, therapeutic relationships, and patient outcomes.

## Appendix

Multimedia Appendix 1 Survey instrument in English and German.

Multimedia Appendix 2 CHERRIES (Checklist for Reporting Results of Internet E-Surveys) checklist.

Multimedia Appendix 3 Journal Article Reporting Standards for Quantitative Research (JARS-Quant) manuscript checklist.

Multimedia Appendix 4 Free-text themes for tools used and purposes of use.

## Abbreviations

aOR: adjusted odds ratio
CHERRIES: Checklist for Reporting Results of Internet E-Surveys
GDPR: General Data Protection Regulation
GenAI: generative AI
JARS-Quant: Journal Article Reporting Standards for Quantitative Research
LLM: large language model
OR: odds ratio

## Data Availability

Deidentified participant-level data are available upon request. The survey instrument is available in Multimedia Appendix 1.
