# Supplementary material for: Generative AI Adoption in Psychotherapy Practice Among Mental Health Professionals: Multicountry Cross-Sectional Survey
Source: JMIR Ment Health. 2026 Sep 30;13:e105052. doi: 10.2196/105052 (PMC13669705; doi:10.2196/105052)
Supplement: Supplementary file 1 [file mental_v13i1e105052_app1.pdf]

Multimedia Appendix 1: Survey Instrument

This supplementary file contains the complete survey instrument (all sections). Section A and Section D are the items relevant to this paper and are reproduced below for reader reference.

Informed Consent

Researcher Names: Charlotte Blease, Jeffrey Cornelius-White, Jens Gaab, Julian Schwarz, John Torous, Josefin Hagström, Antje Frey Nascimento
Institution: Faculty of Psychology, Basel University
Institutional review board number: 017-25-1
Additional Institution: Missouri State University
Institutional Review Board Number: IRB-FY2025-325

You are invited to take part in a research study exploring how mental health professionals—such as psychotherapists, psychologists, counsellors, and psychiatrists—use and perceive generative artificial intelligence (AI) tools in clinical practice. Generative AI tools include platforms such as ChatGPT, Google's Gemini, Microsoft Copilot, and Nuance DAX Copilot, which are increasingly available to clinicians and the public.

Participation is entirely voluntary. Your responses will be fully anonymous—we will not collect your name, contact details, IP address, or any personally identifying information. The information you provide will be stored securely and identified only by a code.

The survey includes questions about your current use of generative AI tools in your clinical practice, your views on how these tools might support or challenge psychotherapy, and basic information about your professional background. Most questions are multiple choice or Likert scale items. One optional open comment question is also included.

The survey will take approximately 5-7 minutes to complete. If at any point you wish to exit the survey, you are free to do so. Completed responses will be included in the analysis; partial responses will be discarded. Your participation will help us better understand how psychotherapists are engaging with emerging AI technologies and contribute to future ethical guidance and professional standards. The findings may be published in a peer-reviewed scientific journal, with only anonymised data reported.

If you have any questions or concerns about the study, please contact:

Professor Jens Gaab at jens.gaab@unibas.ch
Associate Professor Charlotte Blease at charlotte.blease@uu.se
Professor Jeffrey Cornelius-White at jcornelius-white@missouristate.edu

If you have any questions about your rights as a research participant, concerns or complaints about this research, or you want to talk to someone other than the researcher, please contact MSU's Office of Research Administration at (417) 836-5972 or IRB@missouristate.edu. Regular office hours are M-F 8:00 A.M. to 5:00 P.M., after hour messages will be returned the next business day.

To take part in the survey, please confirm that you agree with the following statements:

I have read and understood the information provided about the study, am 18 years or older, and I agree to participate in this survey.

Choose one of the following answers (Yes / No)

Section A: Use of Generative AI in Psychotherapy

- Q1a. Have you ever purposely used generative AI tools to assist you in any aspect of your psychotherapy practice? [Yes / No]
- Q1b. What tools have you used? [ChatGPT / Microsoft Copilot/Bing AI / Bard/Gemini / Claude / Med-PaLM / Nuance DAX / SUKI / HEIDI / Other] (conditional on Q1a = Yes)
- Q1c. For what purpose(s) have you used generative AI tools? [Session note documentation / Generating psychoeducational materials / Treatment planning / Reviewing/summarizing notes / Administrative tasks / Communication with colleagues / Reflective practice or supervision / Other] (conditional on Q1a = Yes)
- Q1d. In general, have these tools reduced your work burdens? [Yes / No] (conditional on Q1a = Yes)
- Q2a. In the last 12 months, has your employer or professional organization encouraged you to use generative AI tools in your work? [Yes / No / Don't know]
- Q2b. In the last 12 months, has your employer or professional organization prohibited you from using generative AI tools in your work? [Yes / No / Don't know]
- Q2c. In the last 12 months, have you participated in any professional training or workshops on the use of generative AI tools? [Yes / No / Don't know]

Section B: Opinions about Generative AI in Psychotherapy

- Q3. To what extent do you agree that generative AI tools will improve your work related to… [Strongly Disagree / Disagree / Somewhat Disagree / Somewhat Agree / Agree / Strongly Agree / Don't Know]
    - Client information gathering
    - Treatment planning
    - Clinical documentation
    - Enhancing therapeutic insights/reflections
    - Supporting clients outside of sessions (e.g., AI-driven journaling or chatbots)
    - Conveying empathy effectively in client communication
    - Understanding yourself to become more congruent with clients
    - Conveying unconditional positive regard effectively in client communication
    - Employing interventions in response to client markers, motivation, or requests
    - Communication with other healthcare professionals
- Q4. To what extent do you agree that the use of generative AI tools in psychotherapy will… [Strongly Disagree / Disagree / Somewhat Disagree / Somewhat Agree / Agree / Strongly Agree / Don't Know]
    - Increase client concerns around confidentiality
- Q5. As a result of generative AI, the meaningfulness of my job to me personally will… [Increase / Not change / Decrease]
- Q6. Please think about how your practice could be affected by generative AI. In your opinion, these tools will: [Decrease my risk of having legal action taken against me / Increase my risk of having legal action taken against me / Neither decrease nor increase my risk / Don't know]
- Q7. What percentage of your patients do you believe currently use generative AI tools for mental health-related purposes? [0% / 1-10% / 11-25% / 26-50% / 51-75% / 76-100% / Unsure]
- Q8. In general, do you believe generative AI tools are helpful or harmful for patients' mental health? [Strongly harmful / Somewhat harmful / Neither harmful nor helpful / Somewhat helpful / Strongly helpful / Unsure]

- Q9. To what extent do you agree with the following statement: "Generative AI will eventually replace most of the work currently done by human therapists." [Strongly Disagree / Disagree / Neither agree nor disagree / Agree / Strongly Agree]
- Q10. How soon do you think generative AI could replace most of the work done by therapists? [Within the next 5 years / Within 10 years / Within 20 years / More than 20 years from now / Unsure] (conditional on Q9 = Agree / Strongly Agree)

Section C: Open-Ended Feedback

- F1. Please add any comments about this topic or the survey. [free-text]

Section D: Demographic Information

- S1. Which of the following best describes your professional role? [multi-category: Private psychotherapist / Psychotherapist employed by a public healthcare system (e.g., NHS, Medicare, national health service) / Psychotherapist in a university or academic setting / Psychotherapist in a nonprofit or NGO / Counsellor / Clinical psychologist / Counselling psychologist / Psychiatrist incorporating psychotherapy in practice / Social worker providing psychotherapy / Mental health nurse providing psychotherapy / Self-employed in mental health services / Other psychologist (please specify below) / Other]
- S1a. Please specify type of psychologist: (conditional on S1 = Other psychologist)
- S2a. Which formats or tools do you currently use in your therapeutic practice? [multi-select: In-person / face-to-face sessions / Phone or video-based sessions (teletherapy) / Asynchronous communication (e.g., email, messaging, chat) / Digital tools or platforms to support therapy (e.g., apps, online exercises, automated check-ins, AI tools)]
- S2b. In the past 3 months, what has been your primary modality for delivering therapy? [Mostly in-person (≥75% of sessions face-to-face) / Mostly teletherapy — live phone or video (≥75% remote) / About an even mix (≈40–60% split between in-person and teletherapy) / Other]
- D1. What is your gender? [Male / Female / Prefer not to say / Other]
- D2. What is your age? [numeric]
- D3. Please specify the country where you work. [free-text]
- D4. Which of the following best describes the area where your practice is based? [Urban / Suburban / Rural]

German version of survey

Informierte Einverständniserklärung

Forschende: Charlotte Blease, Jeffrey Cornelius-White, Jens Gaab, Julian Schwarz, John Torous, Josefin Hagström, Antje Frey Nascimento

Institution: Fakultät für Psychologie, Universität Basel
Institutionelle Überprüfung Board Nummer: 017-25-1
Zusätzliche Institution: Missouri State University
Institutionelles Referenznummer der Ethikkommission: IRB-FY2025-325

Wir laden Sie ein, an unserer Studie teilzunehmen, in der untersucht wird, wie Expert:innen für psychische Gesundheit– Psychotherapeut:innen, Psycholog:innen, Berater:innen und Psychiater:innen generative künstliche Intelligenz (KI) in der klinischen Praxis verwenden und wahrnehmen. Generative KI-Tools umfassen Plattformen wie ChatGPT, Googles Gemini, Microsoft Copilot und Nuance DAX Copilot, Hinzukommt KI-gestützte Dokumentationssoftware, die zunehmend für Behandelnde und die Öffentlichkeit zur Verfügung stehen.

Die Teilnahme ist absolut freiwillig. Ihre Antworten werden vollständig anonym verarbeitet – wir werden weder Ihren Namen, Ihre Kontaktdaten, Ihre IP-Adresse noch personenbezogene Daten erheben. Identifizierung von Informationen: Die von Ihnen bereitgestellten Informationen werden sicher und mit einem anonymisierten Code gespeichert.

Die Umfrage beinhaltet Fragen zu Ihrem aktuellen Einsatz von generativen KI-Tools in Ihrer klinischen Praxis, Ihre Ansichten darüber, wie diese Tools unterstützen können oder eine Herausforderung in der Psychotherapie sind und grundlegende Informationen über Ihren beruflichen Hintergrund. Die meisten Fragen sind im Mehrere-Auswahl- oder Likert-Skala-Format. Offene Fragen, bzw. die Möglichkeit, einen Kommentar zu hinterlassen, sind ebenfalls gegeben.

Das Ausfüllen der Umfrage wird etwa 5-7 Minuten dauern. Wenn Sie die Umfrage an einem Punkt verlassen möchten, können Sie dies jederzeit tun. Wir werden nur vollständig ausgefüllte Umfragen für die Analyse verwenden; Teilantworten werden verworfen. Ihre Teilnahme wird uns helfen, besser zu verstehen, wie Psychotherapeut:innen KI-Technologien verwenden, und Sie tragen damit zur Erstellung künftiger ethischer Leitlinien und Standards bei. Es ist geplant, die Ergebnisse, mit ausschliesslich anonymisierten Daten, in einem peer-reviewten wissenschaftlichen Fachmagazin zu veröffentlichen.

Wenn Sie Fragen oder Bedenken zur Studie haben, wenden Sie sich bitte an:

Professor Jens Gaab bei jens.gaab@unibas.ch
Associate Professor Charlotte Blease bei charlotte.blease@uu.se
Professor Jeffrey Cornelius-White bei jcornelius-white@missouristate.edu

Wenn Sie Fragen zu Ihren Rechten als Forschungsteilnehmer:in haben, Bedenken oder Beschwerden über diese, oder Sie wollen mit jemand anderem sprechen als den genannten Forschenden, wenden Sie sich bitte an das MSU-Büro der Forschungsverwaltung (417) 836-5972 oder IRB@missouristate.edu. Regelmäßige Öffnungszeiten sind M-F 8:00 A.M. bis 5:00 Uhr. Es wird in der Regel am nächsten Werktag geantwortet.

Um an der Umfrage teilzunehmen, bestätigen Sie bitte die folgenden Angaben:

Ich habe die Informationen zur Studie gelesen und verstanden, bin 18 Jahre oder älter und stimme zu, an dieser Umfrage.

Bitte wählen Sie eine der folgenden Antworten [Ja / Nein]

Section A: Verwendung von Generativer KI in der Psychotherapie

- Q1a. Haben Sie jemals gezielt generative KI-Tools verwendet für irgendeinen Bereich Ihrer psychotherapeutischen Tätigkeit? [Ja / Nein]
- Q1b. Welche Werkzeuge haben Sie verwendet? [ChatGPT / Microsoft Copilot / Gemini / Bing AI / Bard / Claude / Perplexity / Consensus / Med-PaLM / NUANCE DAX / SUKI / HEIDI / Sonstiges] (conditional on Q1a = Ja)
- Q1c. Zu welchem Zweck haben Sie generative KI-Tools verwendet? [Dokumentation der Sitzung / Erstellen von psychoedukativen Materialien für Klient:innen / Unterstützung bei der Behandlungsplanung / Überprüfung und Zusammenfassung früherer Sitzungsnotizen / Verwaltung von administrativen Aufgaben (z.B. Terminplanung, Berichtsschreiben) / Kommunikation mit Kolleg:innen verbessern / Zur Unterstützung bei der Reflektion oder Supervision / Sonstiges] (conditional on Q1a = Ja)
- Q1d. Haben diese Hilfsmittel generell Ihre Arbeitsbelastungen reduziert? [Ja / Nein] (conditional on Q1a = Ja)
- Q2a. Hat Ihr Arbeitgeber oder Ihre Organisation Sie in den letzten 12 Monaten die Nutzung generativer KI-Tools in Ihrer Arbeit gefördert? [Ja / Nein / Ich weiss es nicht]
- Q2b. Hat Ihr Arbeitgeber oder Ihre Organisation Ihnen in den letzten 12 Monaten verboten generativen KI-Tools zu nutzen? [Ja / Nein / Ich weiss es nicht]
- Q2c. Haben Sie in den letzten 12 Monaten an einem beruflichen Training oder Workshop zum Einsatz generativer KI Tools teilgenommen? [Ja / Nein / Ich weiss es nicht]

Section B: Einstellungen zur Generativen KI in der Psychotherapie

- Q3. Inwieweit stimmen Sie zu, dass generative KI-Tools Ihre Arbeit verbessern... [Stimme gar nicht zu / Stimme nicht zu / Stimme eher nicht zu / Stimme eher zu / Stimme zu / Stimme sehr zu / Ich weiss es nicht]
    - Erfassung der Daten
    - Behandlungsplanung
    - Klinische Dokumentation
    - Verbesserung therapeutischer Erkenntnisse/Reflexionen
    - Unterstützung von Klient:innen außerhalb der Sitzungen (z.B. durch KI-gestütztes Journaling oder Chatbots)
    - Empathie effektiv in der Kommunikation mit Klient:innen zu nutzen
    - Sich selbst besser zu verstehen, um kongruenter bei Klient:innen zu sein
    - Übermittlung bedingungsloser positiver Wertschätzung in der Kommunikation mit Klient:innen
    - Einsetzen von Interventionen in Reaktion auf patientenseitige Marker, Motivation oder Anfragen
    - Kommunikation mit anderen Fachkräften im Gesundheitswesen
- Q4. Inwieweit stimmen Sie zu, dass der Einsatz generativer KI-Werkzeuge in der Psychotherapie… [Stimme gar nicht zu / Stimme nicht zu / Stimme eher nicht zu / Stimme eher zu / Stimme zu / Stimme sehr zu / Ich weiss es nicht]
    - Bedenken von Klient:innen bezüglich Vertraulichkeit verstärkt
- Q5. Infolge generativer KI wird sich die Sinnhaftigkeit meiner Arbeit für mich persönlich... [Erhöhen / Nicht ändern / Abnehmen]
- Q6. Denken Sie bitte darüber nach, wie Ihre klinische Arbeit durch generative KI beeinflusst werden könnte. Ihrer Meinung nach werden diese Hilfsmittel: [Das Risiko verringern, dass rechtliche Schritte gegen mich unternommen werden / Das Risiko erhöhen, dass rechtliche

Schritte gegen mich unternommen werden / Das Risiko weder verringern noch erhöhen / Ich weiss es nicht]

- Q7. Was glauben Sie, welcher Prozentsatz Ihrer Patient:innen nutzt aktuell generative KI-Tools zur psychischen Gesundheit? [0% / 1-10% / 11-25% / 26-50% / 51-75% / 76-100% / Unsicher]
- Q8. Glauben Sie, dass generative KI-Werkzeuge für die psychische Gesundheit von Patient:innen grundsätzlich hilfreich oder schädlich sind? [Sehr schädlich / Etwas schädlich / Weder schädlich noch hilfreich / Etwas hilfreich / Sehr hilfreich / Unsicher]
- Q9. Inwiefern stimmen Sie mit der folgenden Erklärung überein: "Generative KI könnte irgendwann den größten Teil der Arbeit ersetzen, die derzeit von Therapeut:innen geleistet wird." [Ich stimme gar nicht zu / Ich stimme nicht zu / Neutral / Ich stimme zu / Ich stimme absolut zu]
- Q10. Wie bald denken Sie, könnte generative KI einen Großteil der Arbeit von Therapeut:innen ersetzten? [Innerhalb der nächsten 5 Jahre / Innerhalb von 10 Jahren / Innerhalb von 20 Jahren / Nach über 20 Jahren ab jetzt / Unsicher] (conditional on Q9 = Ich stimme zu / Ich stimme absolut zu)

Section C: Open-Ended Feedback

- F1. Bitte fügen Sie hier jegliche Kommentare zum Thema oder der Umfrage hinzu. [Freitext]

Section D: Demographische Informationen

- S1. Welche der Folgenden beschreibt Ihr berufliche Rolle am besten? [Psychotherapeut:in privat tätig / Psychotherapeut:in, im öffentlichen Gesundheitssystem tätig / Psychotherapeut:in an einer Universität oder akademischen Umgebung / Psychotherapeut:in in einer gemeinnützigen Institutionoder NGO / Berater:in / Klinische Psycholog:inn / Psycholog:in in Beratung tätig / Psychiater:in, die/der Psychotherapie anbietet / Sozialarbeiter:in, die/der Psychotherapie anbietet / Therapieanbietende:r Krankenpfleger:in für psychische Gesundheit / Selbständige:r, die/der im psychischen Gesundheitsdiensten tätig / Andere:r Psycholog:in (bitte unten angeben) / Sonstiges]
- S1a. Bitte spezifizieren Sie die Art von Psychotherapeut:in die/der Sie sind. [Freitext] (conditional on S1 = Andere:r Psycholog:in (bitte unten angeben))
- S2a. Welche Formate oder Tools verwenden Sie derzeit in Ihrer therapeutischen Praxis? [Vor Ort / Face-to-face Sitzungen / Telefon- oder Video-basierte Sitzungen (Teletherapie) / Asynchrone Kommunikation (z.B. E-Mail, Nachrichten, Chat) / Digitale Tools oder Plattformen zur Unterstützung der Therapie (z.B. Apps, Online-Übungen, automatisierte Check-Ins, KI-Tools)] (multi-select)
- S2b. In welcher Form fanden Ihre Therapiesitzungen in den letzten 3 Monaten überwiegend statt? [Meist persönlich (≥75 % der Sitzungen face-to-face) / Meist Teletherapie — Telefonat oder Video (≥75% remote) / Eine gleichmässige Mischung (≈40–60% Aufteilung zwischen In-Person und Teletherapie) / Sonstiges]
- D1. Was ist Ihr Geschlecht? [Männlich / Weiblich / Keine Angabe / Sonstiges]
- D2. Was ist Ihr Alter? [numerisch]
- D3. Bitte geben Sie an, in welchem Land Sie arbeiten. [Freitext]
- D4. Welche der folgenden Optionen beschreibt den Standort Ihrer Praxis am besten? [Stadt / Vorstadt / Land]
