# Supplementary material for: Generative AI Adoption in Psychotherapy Practice Among Mental Health Professionals: Multicountry Cross-Sectional Survey
Source: JMIR Ment Health. 2026 Sep 30;13:e105052. doi: 10.2196/105052 (PMC13669705; doi:10.2196/105052)
Supplement: Supplementary file 2 [file mental_v13i1e105052_app2.pdf]

Multimedia Appendix 2. Checklist for Reporting Results of Internet E-Surveys (CHERRIES)

Item Category	Checklist item	Described in the manuscript	Cited from the manuscript
**Design**	Describe survey design	Yes	In **Methods > Study Design**
**IRB (Institutional Review Board) approval and informed consent process**	IRB approval	Yes	**Methods > Ethical considerations**<br>Ethical approval was obtained from the Institutional Review Board of the Faculty of Psychology, University of Basel (IRB number: 017-25-1) and Missouri State University's Institutional Review Board (IRB-FY2025-325).
	Informed consent	Yes	**Methods** > **Ethics Approval**<br>All participants provided informed consent prior to participation.
	Data protection	Yes	**Methods** > **Ethics Approval**<br>Data were collected using LimeSurvey, a secure online platform that employs encryption and anonymization to prevent survey responses from being linked to individual participants. All identifying information, including email addresses, was removed prior to the dataset being shared with the research team. The platform operates in compliance with the European Union General Data Protection Regulation (GDPR).
**Development and pre-testing**	Development and testing	Yes	**Methods > Survey Instrument**<br>The survey was developed iteratively by the lead members of the research team (CB, J C-W, and JG) with expertise in digital mental health, clinical psychology and psychotherapy, and survey methodology. Items were piloted with a convenience sample of three practicing clinicians from the US, UK, and Sweden before finalization.
**Recruitment process and description of the sample having access to the questionnaire**	Open survey versus closed survey	Yes	**Methods > Recruitment**<br>The survey link was disseminated directly and through snowball-sampling requests to professional contacts. Recruitment channels included professional associations and networks (psychology, psychiatry, counselling, and social work bodies), academic and clinical mailing lists. We deliberately avoided use of all social media platforms to administer the survey to avoid the risk of bot contamination.
	Contact mode	Yes	**Methods > Recruitment**

			The survey link was disseminated directly and through snowball-sampling requests to professional contacts via e-mail. Recruitment channels included professional associations and networks (psychology, psychiatry, counselling, and social work bodies), academic and clinical mailing lists.
	Advertising the survey	Yes	**Methods > Recruitment**<br>The survey link was disseminated directly and through snowball-sampling requests to professional contacts via e-mail. Recruitment channels included professional associations and networks (psychology, psychiatry, counselling, and social work bodies), academic and clinical mailing lists.
**Survey administration**	Web/E-mail	Yes	**Methods > Recruitment**<br>The survey link was disseminated directly and through snowball-sampling requests to professional contacts via e-mail.
	Context	Yes	**Methods > Recruitment**<br>Participants were recruited via a global convenience sampling strategy between 13 January 2026 and 30 March 2026 (approximately 11 weeks). The survey link was disseminated directly and through snowball-sampling requests to professional contacts via e-mail. Recruitment channels included professional associations and networks (psychology, psychiatry, counselling, and social work bodies), academic and clinical mailing lists.
	Mandatory/voluntary	Yes	**Methods > Ethics Approval**<br>All participants provided informed consent prior to participation.
	Incentives	Yes	**Methods > Ethics Approval**<br>Participation was voluntary, anonymous, and unpaid.
	Time/Date	Yes	**Methods > Recruitment**<br>Participants were recruited via a global convenience sampling strategy between 13 January 2026 and 30 March 2026 (approximately 11 weeks).
	Randomization of items or questionnaires	No	**--**
	Adaptive questioning	Yes	**Methods > Recruitment**<br>Conditional branching was used for selected items.
	Number of Items	Yes	**Methods > Survey Instrument**<br>Section A — AI Use Behaviors (7 items)<br>Section D — Demographics (7 items)
	Number of screens (pages)	Yes	**Methods > Survey Instrument**<br>Participants

	Completeness check	Yes	**Methods > Survey Instrument**<br>The questionnaire was presented group-by-group in LimeSurvey and consisted of five survey sections/pages.
	Review step	No	
**Response rates**	Unique site visitor	No	Number not provided by LimeSurvey.
	View rate (Ratio of unique survey visitors/unique site visitors)	No	Number not provided by LimeSurvey.
	Participation rate (Ratio of unique visitors who agreed to participate/unique first survey page visitors)	Yes	**Results > Sample Characteristics**<br>The participation rate (unique visitors who agreed to participate / unique first survey page visitors) was 1,052 /1,116 (94.26%).
	Completion rate (Ratio of users who finished the survey/users who agreed to participate)	Yes	**Results > Sample Characteristics**<br>A total of 766 participants completed the survey (completion rate: 72.6% of 1055 who opened the survey link).
**Preventing multiple entries from the same individual**	Cookies used	Yes	**Methods > Recruitment**<br>To preserve respondent anonymity, IP addresses, cookies, or registration identifiers were not collected.
	IP check	Yes	**Methods > Recruitment**<br>Same as above.
	Log file analysis	Yes	**Methods > Recruitment**<br>Same as above.
	Registration	Yes	**Methods > Recruitment**<br>Same as above.
**Analysis**	Handling of incomplete questionnaires	Yes	**Methods > Statistical Analysis**<br>Only completed questionnaires were included in the analyses.
	Questionnaires submitted with an atypical timestamp	Yes	**Methods > Recruitment**<br>No timestamp-based duplicate-response screening was undertaken.
	Statistical correction	No	--

Reference:

Eysenbach G. Improving the quality of Web surveys: the Checklist for Reporting Results of Internet E-Surveys (CHERRIES). J Med Internet Res 2004;6:e34.
