# Supplementary material for: Generative AI Adoption in Psychotherapy Practice Among Mental Health Professionals: Multicountry Cross-Sectional Survey
Source: JMIR Ment Health. 2026 Sep 30;13:e105052. doi: 10.2196/105052 (PMC13669705; doi:10.2196/105052)
Supplement: Supplementary file 3 [file mental_v13i1e105052_app3.pdf]

Appendix 4

JARS–Quant Manuscript Checklist

Section	Checklist Item	Reported	Comment
Title and Title Page	Title	Yes	
Title and Title Page	Identify main variables and theoretical issues under investigation and relationships between them.	Yes	
Title and Title Page	Identify the populations studied.	Yes	
Title and Title Page	Author Note: registration information, prior reporting, funding/support, conflicts, affiliations, corresponding author, and other important information.	Yes	
Abstract	Objectives: State the problem under investigation, including main hypotheses.	Yes	
Abstract	Participants: Describe participants and pertinent characteristics.	Yes	
Abstract	Study Method: Describe design, sample size, materials, outcome measures, and data-gathering procedures.	Yes	
Abstract	Findings: Report findings, including effect sizes and confidence intervals or statistical significance levels.	Yes	
Abstract	Conclusions: State conclusions, implications, or applications beyond just results.	Yes	
Introduction	Problem: State the importance of the problem, including theoretical or practical implications.	Yes	
Introduction	Review of Relevant Scholarship: Review relevant scholarship and relation/differences to previous work.	Yes	
Introduction	Hypotheses, Aims, and Objectives: State specific hypotheses, aims, objectives, theories, planned analyses, and how hypotheses relate to design.	Yes	
Method	Inclusion and Exclusion: Report inclusion/exclusion criteria and demographic restrictions.	Yes	
Method	Participant Characteristics: Report major demographic and important topic-specific characteristics.	Yes	
Method	Sampling Procedures: Describe participant selection, sampling method, participation/self-selection, setting/location, dates, agreements/payments, and ethics.	Yes	
Method	Sample Size, Power, and Precision: Report intended/achieved sample size and how sample size or precision was determined.	Yes	
Method	Measures and Covariates: Define all primary/secondary measures and covariates, including collected measures not included in the report.	Yes	
Method	Data Collection: Describe methods used to collect data.	Yes	
Method	Quality of Measurements: Describe methods used to enhance measurement quality, including training/reliability and multiple observations.	Partial	Pilot testing reported, but no extensive interviewer/training procedures relevant to this survey.
Method	Instrumentation: Provide information on validated or ad hoc instruments and relevant psychometric/biometric properties.	Partial	Survey development described, no psychometric validation conducted.
Method	Masking: Report awareness of condition assignments and how masking was accomplished/evaluated, if applicable.	N/A	Not relevant to observational survey.
Method	Psychometrics: Report reliability and relevant convergent/discriminant validity estimates.	N/A	Not applicable for this descriptive survey.
Method	Conditions and Design: State whether conditions were manipulated or naturally observed and report the design type.	Yes	
Method	Data Diagnostics: Describe exclusion criteria, missing-data procedures, outlier processing, distribution analyses, and transformations.	Partial	
Method	Analytic Strategy: Describe inferential analytic strategy and protection against experiment-wise error for primary, secondary, and exploratory hypotheses.	Yes	
Results	Participant Flow: Report participant numbers and flow through each stage; include a flow figure when possible.	Yes	Flow chart in Figure 1.
Results	Recruitment: Provide dates for recruitment and repeated measures/follow-up.	Yes	
Results	Statistics and Data Analysis: Report missing data, outcome descriptions, inferential statistics, effect sizes/confidence intervals, and distinguish primary, secondary, and exploratory analyses.	Yes	
Results	Complex Data Analyses: Report model details, relevant variance-covariance/correlation matrices, and statistical software used.	Yes	

Results	Estimation Problems/Anomalies: Report convergence problems, diagnostics, anomalies, and solutions.	Yes	
Results	Other Data Analyses: Identify adjusted analyses and indicate which were planned versus unplanned.	Yes	
Results	Statistical Assumptions: Report problems with assumptions or data distributions that could affect validity.	Partial	
Discussion	Support of Original Hypotheses: State support/nonsupport for all primary and secondary hypotheses and discuss exploratory analyses.	Partial	Research objectives discussed, however not framed as formal hypotheses.
Discussion	Similarity of Results: Discuss similarities and differences between reported results and other work.	Yes	
Discussion	Interpretation: Interpret results considering bias, validity, measurement imprecision, multiple/overlapping tests, sample size, and sampling validity.	Yes	
Discussion	Generalizability: Discuss external validity considering target population and contextual issues.	Yes	
Discussion	Implications: Discuss implications for future research, programs, or policy.	Yes	
