# Supplementary material for: Generative AI Adoption in Psychotherapy Practice Among Mental Health Professionals: Multicountry Cross-Sectional Survey
Source: JMIR Ment Health. 2026 Sep 30;13:e105052. doi: 10.2196/105052 (PMC13669705; doi:10.2196/105052)
Supplement: Supplementary file 4 [file mental_v13i1e105052_app4.pdf]

Multimedia Appendix 3: Free-Text Themes for "Tools Used" and "Purposes of Use"

Table S1. Other generative AI tools mentioned in free-text responses and frequencies (question Q1b).

**Tool**	**n**
Le Chat (Mistral)	7
Grok	3
PlaynVoice	3
Locally developed AI tool	3
Zoom AI Companion	2
Gamma	2
Otter AI	2
NotebookLM	2
Proton Lumo	2
Embedded genAI note-taking	2
Alpine AI	1
Aurora	1
BerichtBiber	1
Canva	1
Carelog	1
Terapeutbooking	1
DeepAI image generator	1
DeepL	1
Duck AI	1
Coachin	1
Elicit	1
Epic	1
Epikur KI	1
Grammarly	1
Klarify	1
Lindy	1
Logicc	1
Neuroflash	1
NOA (Jameda)	1
Open Evidence	1
Plaud	1
Scite.AI	1

SpeechAI	1
Teams AI	1
Upheal	1
Voilà	1
Wispr Flow	1
Non–AI-based applications (PVS System Tomedo, PDF, Dictation function in Microsoft Word)	3
“Different ones without login”	1
Don’t remember	1

Table S2. Other purposes of AI tool use mentioned in free-text responses, examples, and frequencies (Q1c).

**Theme**	**Examples**	**n**
Text production and language	Writing emails, blogs, reports, articles, etc	27
	Translation	
	Spelling, grammars	
	Formatting	
	Client communication	
Information search	Evidence for treatment	17
	Methods	
	Medical conditions	
	Technical terms and topics	
	Learning about client's cultural background	
	Clarification of legal issues	
Professional reporting	Case reports	15
	Protocol	
	Report (discharge)	
	Expert opinion	
Clinical reasoning	Explaining confidentiality	13
	Diagnostics	
	Case formulation	
	Test evaluation	
Teaching/training	Presentations	7
	Planning seminar structure	
	Assignments	
Material creation	Psychoeducational materials	6
	Script for explanation of confidentiality to clients	
	Creating treatment plans	
	Exercises	
	Graphics for client homework	
	Creating HIPAA-compliant forms	
Creativity	Inspiration	6
	Marketing	
	Social media posts	
	Ideas for therapy check-in and check-out tasks	
Administrative tasks	Organizing notes	4
	Creating school schedule	
	HIPAA form (legal requirements)	
Research support	Generating keyword terms/tags for research articles	3
	Reading suggestions and bibliography	
Technical support	Troubleshooting	3
	Structuring files	
Reflection	Reflection buddy	2
	Reflecting together with clients on their use of AI	
